# Evaluating the clinical potential of bioceramic-rods for revascularization in osteonecrosis of the femoral head: a systematic review

**DOI:** 10.1007/s00264-024-06366-3

**Published:** 2024-11-14

**Authors:** Xiao Lu, Yajie Lu, Zhen Wang, Fangchun Jin, Yicheng Wang, Jianxi Lu

**Affiliations:** 1 Shanghai Orthopedic Biomaterial Technology Innovation Center, Shanghai, China; 2 Xijing Hospital of the Chinese Air Force Medical University, Xian, China; 3 Xin Hua Hospital Affiliated to Shanghai Jiao Tong University School of Medicine, Shanghai, China; 4 Shanghai Sixth People’s Hospital Affiliated to Shanghai Jiao Tong University School of Medicine, Shanghai, China

**Keywords:** Bioceramic Rods, Osteonecrosis of the Femoral Head, Revascularization, Hip Preservation, Clinical Effectiveness

## Abstract

**Objective:**

To evaluate the safety, reliability, and effectiveness of bioceramic rods (BR) in treating osteonecrosis of the femoral head (ONFH), compared with other treatments such as core decompression and autologous bone grafting.

**Design:**

Systematic review and meta-analysis.

**Data sources:**

Pubmed, Embase, and CNKI databases from January 2011 to July 2023.

**Eligibility criteria for study selection:**

Included studies involved patients treated with bioceramic rods. Studies were required to have a follow-up time of more than six months and no statistically significant differences in baseline information between groups in controlled studies. Exclusions included literature reviews, case reports, conference abstracts, animal experiments, and studies without defined success criteria or lacking analysis on factors influencing efficacy.

**Main outcome measures:**

The primary outcome measure was the Harris Hip Score (HHS) improvement rate. Secondary outcomes included the femoral head stability and survival rate, alongside the hip replacement rate.

**Results:**

The systematic review revealed significant improvements in symptom relief and functional recovery using BR for the treatment of ONFH. An average follow-up of 20.44 months showed an overall HHS improvement rate of 33.93%. Hip preservation efficacy with BR was superior to core decompression and autologous bone grafting. The overall femoral head survival rate was 84.42%, with results sustained for three years. The success rate of hip preservation was notably higher with early intervention, which showed better outcomes when the overall HHS improvement exceeded 27%, and rates of excellent and good outcomes approached 90%.

**Conclusions:**

Bioceramic rods offer a safe, minimally invasive, reliable, and effective treatment option for ONFH, ensuring substantial symptom relief and functional recovery. The technique’s success in early disease stages suggests a strong potential for broader clinical adoption. Although additional benefits from combining BR with stem cells, platelet-rich plasma, and traditional Chinese medicine are noted, definitive conclusions on enhanced therapeutic effects remain inconclusive.

## Introduction

**Fig. 1:**
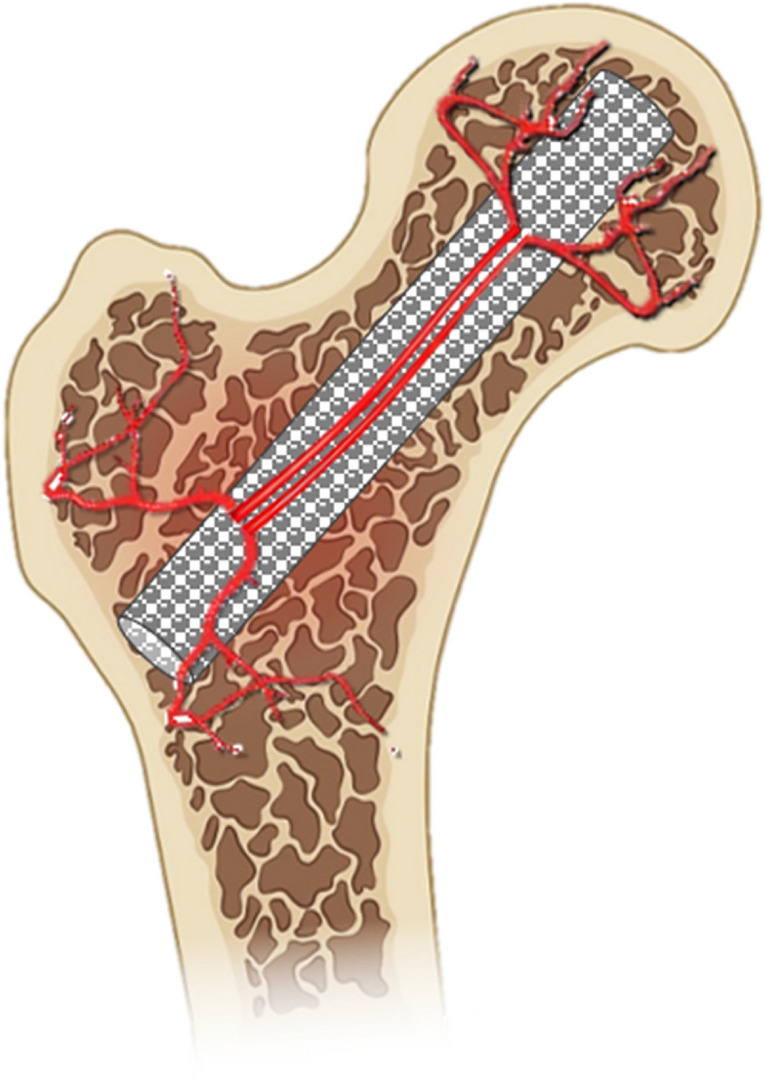
The mechanism of action of bioceramic rod mediated revascularization

Osteonecrosis of the femoral head (ONFH) results from complex vascular disorders, causing tissue necrosis accompanied by intraosseous hypertension, leading to joint destruction. Traditional treatments focus on pain relief and structural support, lacking comprehension of the crucial three-dimensional spatial structure needed for revascularization. Our prior research emphasized the role of bioceramic microstructure in vascular and bone regeneration, revealing the pivotal role of interconnecting pores [1]. Building on this, we introduced the bioceramic rod (BR) revascularization technique, employing porous BR to connect blood supply between the trochanter and head. This redirects blood flow, reconstructing circulation in the affected area (Fig. 1) [2], demonstrating substantial clinical efficacy. A systematic review of relevant clinical studies enhances our understanding, aiming to establish a robust theoretical foundation and effective strategies for widespread application.

## Materials and methods

### Literature retrieval

A comprehensive literature search spanning January 2011 to July 2023 was executed across PubMed, Embase, and CNKI databases. The search employed keywords “bioceramic rod” and “osteonecrosis of the femoral head,” encompassing both ischaemic and aseptic occurrences of femoral head osteonecrosis.

### Selection criteria

#### Inclusion criteria

- Patients with relevant information, an average follow-up time of ≥ six months, and no statistically significant differences in baseline information between the two groups in controlled studies.
- Confirmed cases undergoing treatment with BRs.
- Primary outcome measures include the Harris Hip Score (HHS) and surgical details.
- Efficacy indicators comprise the HHS improvement rate, femoral head survival rate, hip joint excellent, stability, and replacement rates.

#### Exclusion criteria

- Clinical studies unrelated to BRs.
- Absence of number of hips and definition of success criteria.
- Lack of analysis on factors influencing efficacy.
- Literature reviews, case reports, conference abstracts, animal experiments, editorial guidelines or expert comments

### Literature evaluation

The quality assessment of case series studies adhered to the 2011 Oxford Centre for Evidence-Based Medicine (EBM) criteria. Controlled trials were appraised using the modified Jadad scale. Evaluation of the retrieved literature involved independent assessment by two researchers. In instances of divergent opinions, consensus was reached through discussion or the involvement of a third party to assess and determine the risk of bias.

### Data extraction

#### Literature information

Essential information, including the first author, journal title, publication year, study design, language, journal impact factor, citation rate, and view count, was compiled. The impact factor was retrieved from JustScience and 360Net, citation rates were obtained from Google Scholar and CNKI, and view counts were sourced from Baidu and CNKI.

#### Case data

Data on the case count, number of joints, age, gender, aetiology, disease duration, affected joints, diagnostic classification, and follow-up duration were collected.

#### Surgical implants

Surgical implants employed β-tricalcium phosphate with a purity ≥ 95%, manufactured using patented technique [3, 4] into controlled porous bioceramics (Shanghai Bio-Lu Biomaterials Co., Ltd.). This includes BR (φ10 × 60–100 mm) and particles (φ2-3.5 mm), featuring porous structure, macropores of 500–600 μm, interconnections of 120 ± 50 μm, and 65 ± 5% porosity.

#### Surgical procedure

**Fig. 2:**
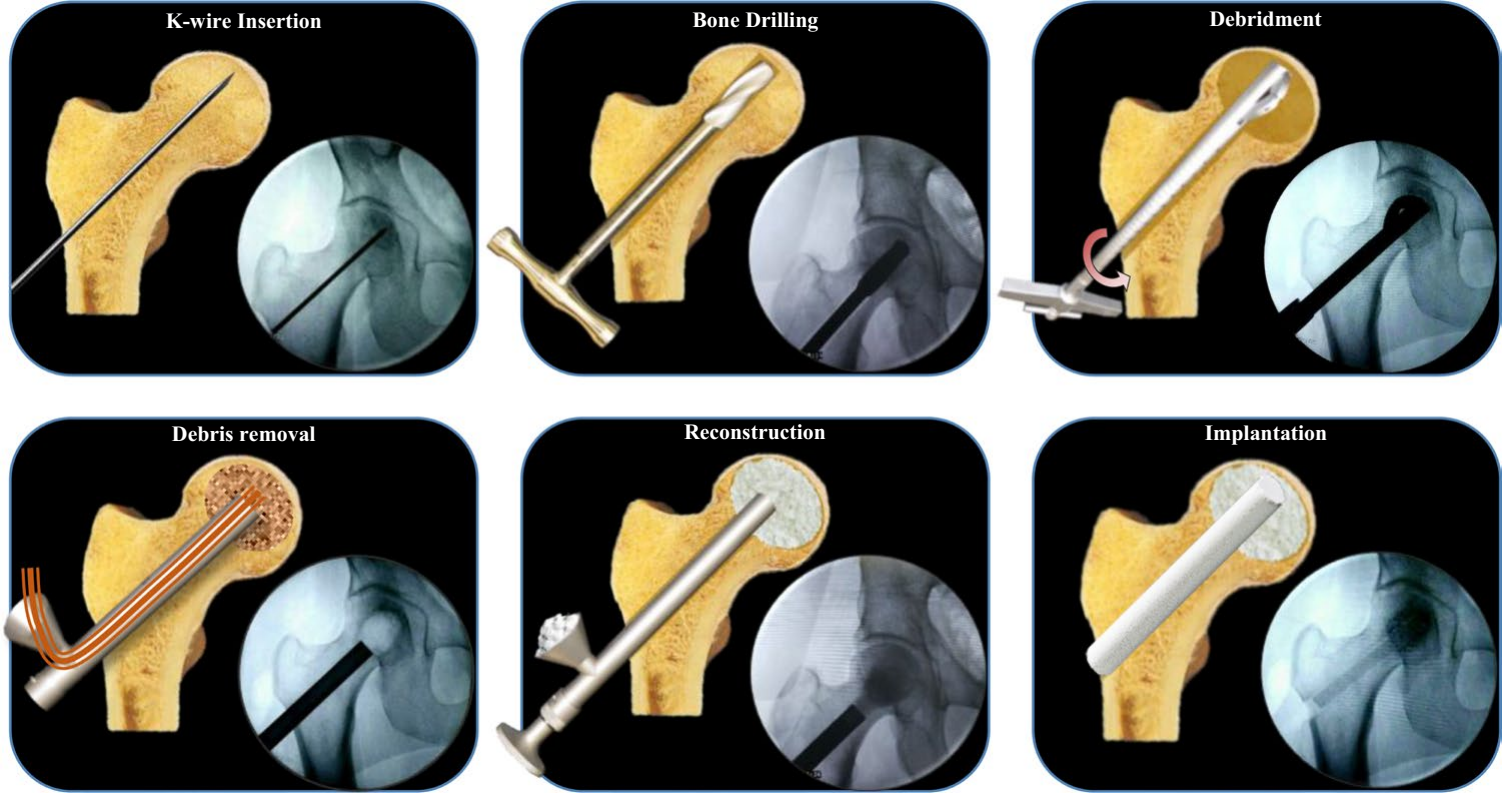
The surgical procedure of bioceramic rod technique

Initially, a K-wire is placed from the greater trochanter to the midpoint between the pubic symphysis and anterior superior iliac spine, reaching the subchondral bone. After confirming its position, a bone incision is made, and a protective sleeve inserted. Using a 12 mm cannulated drill bit, a bony channel is created 5 mm beneath the subchondral bone, followed by necrotic bone removal with an expandable reamer. Debrided tissues are irrigated, suctioned, and implants inserted (Fig. 2). Procedure details, incision length, blood loss, and hospitalization time are meticulously recorded.

#### Data extraction

- Follow-up intervals were categorized by years: ≤1, ≤ 2, ≤3, ≤ 4, ≤5, and ≤ 6 years, corresponding to 3–12, > 12–24, > 24–36, > 36–48, > 48–60, and > 60–72 months.
- Diagnoses followed Association Research Circulation Osseous (ARCO) staging. Two articles [5, 6] utilized the Steinberg classification, which was then converted to ARCO staging for statistical analysis.
- HHS was classified into four categories: excellent (90–100), good (80–90), fair (70–80), or poor (< 70). Calculations were derived from the score:

$$\mathrm{HHS}\;\mathrm{Improvement}\;\mathrm{rate}\;(\mathrm{HIR})=\;(\mathrm{Postoperative}\;\mathrm{HHS}\;-\;\mathrm{Preoperative}\;\mathrm{HHS})\;/\;\mathrm{Preoperative}\;\mathrm{HHS}$$

$$\mathrm{Hip}\;\mathrm{excellent}\;\&\;\mathrm{good}\;\mathrm{rate}\;(\mathrm{HGR})=\;(\mathrm{Excellent}\;\mathrm{hips}\;+\;\mathrm{Good}\;\mathrm{hips})\;/\;\mathrm{Total}\;\mathrm{hips}$$

d) Femoral head stability and survival rates were calculated based on the number of heads demonstrating radiographic improvement, stability, aggravation and severe collapse:

$$\mathrm{Stability}\;\mathrm{rate}\;(\mathrm{HSR})=\;(\mathrm{Improved}\;\mathrm{heads}\;+\;\mathrm{Stable}\;\mathrm{heads})\;/\;\mathrm{Total}\;\mathrm{heads}$$

$$\mathrm{Survival}\;\mathrm{rate}\;(\mathrm{FSR})\;=\;(\mathrm{Total}\;\mathrm{heads}\;-\;\mathrm{Severely}\;\mathrm{collapsed}\;\mathrm{heads})\;/\;\mathrm{Total}\;\mathrm{heads}$$

e) Hip replacement rate was calculated as follows: 

Replacement rate (HRR) = Number of hip replacements / Total hips

f) Postoperative ambulation time, duration of double then single crutch use were documented.

### Statistical analysis

The data underwent mean and standard deviation calculations for primary and secondary indicators. Descriptive analysis was applied to unmergeable data. Statistical analyses were performed using SPSS 20.0 software, employing paired t-tests for pre/postoperative comparisons and independent samples t-tests for inter-group differences. Meta-analysis was conducted using the “Meta” package in R statistical software (version 3.6.2, MathSoft, Massachusetts). Heterogeneity among studies was assessed using the Q-Tet and I^2^ tests (*p* < 0.1 and I^2^ > 50%). When heterogeneity existed, a fixed-effects model was used for pooling analysis; otherwise, a random-effects model was used. Sensitivity analysis was performed when ≥ 9 studies were available by systematically excluding each study from the Meta-analysis to evaluate the stability and credibility of the summary results. Scatter plots were generated using R software, with one set of data on the x-axis and the other on the y-axis. Pearson correlation coefficients were calculated to quantify the linear relationship between the two sets of data (ranging from − 1 to 1). Additionally, the coefficient of determination (R-squared) was calculated as a measure of how well a regression model fits the observed data and was used as an indicator of the overall correlation. Significance was set at *P* < 0.05 for all analyses.

## Results

### Literature assessment

**Fig. 3:**
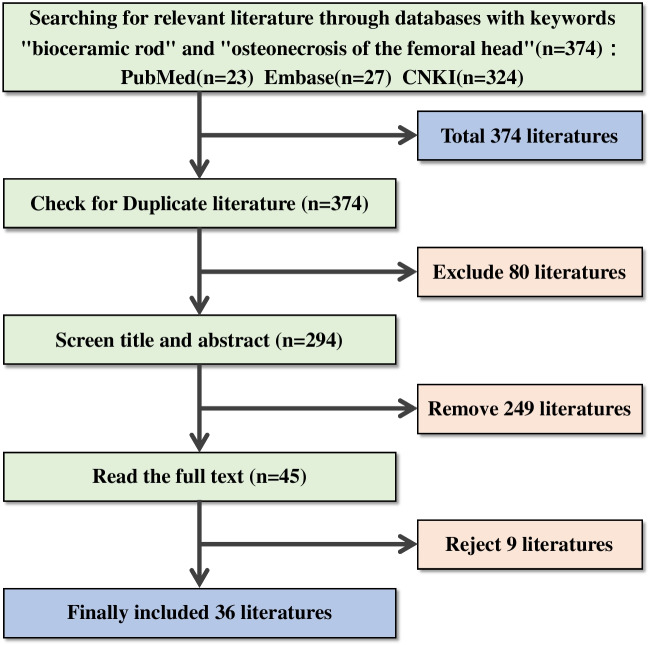
The literature retrieval strategy and process

**Fig. 4:**
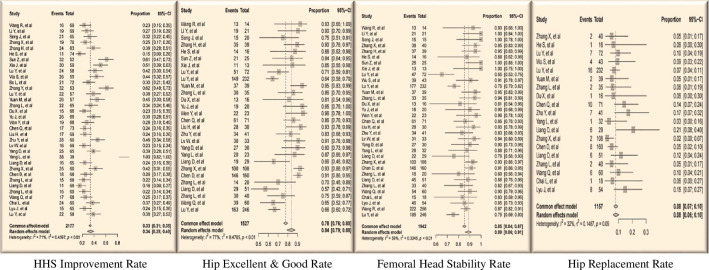
Evaluation results of the sensitivity and stability of core data

Initially, 374 articles were retrieved, with 80 duplicates and 249 exclusions during title and abstract screening. Full-text screening eliminated nine more articles, leaving 36 for inclusion (Fig. 3). In six randomized controlled trials, all showed low selection bias. Among six articles lacking reported allocation concealment, 85.71% had low bias risk due to blinding during intervention. In four articles with blinding in outcome assessment, only 10.53% had low bias risk. All 36 studies had comprehensive outcome data, indicating minimal follow-up bias. Safety/efficacy data across the literature showed a low risk of reporting bias (100%). In 16 controlled studies, 14 revealed no statistically significant baseline differences, minimizing confounding bias. Meta-analysis funnel plot and Egger’s test (*P* > 0.05) suggested no publication bias (Fig. 4). Robust literature indicators, low bias susceptibility, heightened sensitivity, and high homogeneity collectively establish a robust foundation for this study.

The 36 articles, from 28 journals, averaged an impact factor of 2.303 ± 3.501 (maximum 18.9). English articles constituted 19.44%, while 80.56% were in Chinese. Mean citations and views were 5.94 ± 6.73 (maximum 30) and 139.61 ± 72.26 (maximum 304), respectively. 61.11% received research fund support. The literature offered reliable, readable, practical, and impactful information and data.

### Case data

From October 2011 to May 2022, 1965 ONFH patients (2222 hips) were treated, mean age 40.01 ± 4.90 years (16–70 years), with a disease duration of 10.52 ± 6.57 months (0.5–48 months). Unilateral cases dominated at 87.56%, significantly higher (7.04 times) than bilateral (12.44%) (*P* = 0.00004). Males comprised 66.21%, twice the female rate (33.79%) (*P* = 0.00015). Etiological factors included alcohol consumption (39.35%), steroid use (34.05%), idiopathic causes (20.75%), and trauma (5.85%). Alcohol and steroids were significantly higher than idiopathic and trauma (*P* < 0.05). The ARCO staging distribution was as follows: I (11.12%), II (76.33%), III (12.47%), and IV (0.09%) (Table 1). The study primarily focuses on middle-aged individuals in stage II of ONFH.

**Table 1:** Included literature and baseline case information

*N*_O_	Author	Year	Study	Language	Gender		Age (years)	Hip	ARCO Stage (hip)				Follow-up (months)	Treatment	Read (times)
					M	F			I	II	III	IV			
1	Wang R	2015	CS	Chi.	5	8	45.3	14	6	8	0	0	17	CD + CR	273
2	Li YC	2016	CS	Chi.	11	6	40.6	21	6	15	0	0	24	CD + CR + ESC	160
3	Song J	2017	CS	Chi.	10	15	42.5	25	8	17	0	0	36	CD + CR	141
4	Zhang XD	2017	CS	Chi.	26	6	39.0	42	7	35	0	0	24	CD + CR	98
5	Zhang HL	2017	CS	Chi.	30	9	38.0	39	0	39	0	0	16	CD + CR	131
6	He SM	2017	CS	Chi.	4	8	31.5	16	1	9	6	0	19	CD + CR	182
7	Sun Z	2017	CS	Chi.	18	5	41.3	25	0	25	0	0	13	CD + CR	210
8	Xie JB	2017	CS	Chi.	3	8	46.5	13	6	5	2	0	16	CD + CR	112
9	Lu YJ	2018	CS	Eng.	45	17	44.5	72	0	43	29	0	27	CD + CR	123
10	Wu SC	2018	CC	Chi.	24	18	36.8	45	26	19	0	0	18	CR vs. CR + TCM	143
11	Li B	2018	CC	Eng.	25	15	41.0	40	13	14	13	0	18	CD + CR	102
12	Zhang YX	2019	CS	Chi.	36	10	41.3	46	0	46	0	0	12	CD + CR	157
13	Lu YJ	2019	CS	Chi.	145	55	42.0	232	0	150	82	0	23	stage II vs. III	258
14	Yuan MW	2019	RCT	Chi.	25	14	43.0	49	0	49	0	0	18	CR vs. CR + PRP	251
15	Zhang LL	2019	CC	Chi.	22	10	33.2	35	0	33	2	0	15	CR vs. CR + TCM	132
16	Du XG	2019	CS	Chi.	11	2	36.3	16	7	9	0	0	24	CR vs. CR + PRP	96
17	Yu JW	2019	RCT	Chi.	18	14	35.4	40	13	27	0	0	28	CR vs. CD	119
18	Wen Y	2019	CS	Chi.	15	6	36.0	23	3	20	0	0	16	CD + CR	51
19	Chen Q	2019	CS	Chi.	49	13	40.6	71	10	61	0	0	14	CD + CR	115
20	Liu H	2019	CS	Chi.	19	11	45.2	30	12	18	0	0	12	CD + CR	69
21	Zhu YK	2020	CC	Chi.	23	9	35.0	41	0	41	0	0	12	CR vs. CR + TCM	77
22	Lu WH	2020	CC	Chi.	32	28	42.6	67	31	36	0	0	12	CR vs. ABG	111
23	Yang DG	2020	CS	Chi.	16	14	52.8	30	0	30	0	0	12	CD + CR	64
24	Yang LB	2020	CS	Chi.	18	5	29.5	32	0	23	9	0	24	CD + CR	95
25	Liang DW	2020	CS	Chi.	19	10	43.0	29	0	17	12	0	25	CD + CR	171
26	Zhang X	2020	RCT	Chi.	97	11	42.2	108	61	47	0	0	24	CR vs. CR + PRP	144
27	Wang XO	2020	CS	Chi.	38	32	42.5	70	0	62	8	0	6	CD + CR	50
28	Chen DD	2020	CC	Chi.	55	45	44.6	160	0	160	0	0	12	CR vs. CR + PRP	298
29	Zhang LL	2021	CC	Eng.	21	15	34.0	39	0	31	8	0	29	CR vs. GAB	88
30	Li QT	2021	RCT	Eng.	31	9	37.7	57	3	43	9	2	24	CR vs. ESC	109
31	Zhang LL	2021	CC	Chi.	23	17	35.0	42	0	34	8	0	26	CR vs. AB	171
32	Wang Q	2022	CC	Eng.	23	31	33.2	60	0	60	0	0	12	CR vs. CR + PRP	/
33	Chai L	2022	CC	Chi.	23	14	37.0	37	21	16	0	0	18	CR vs. AB	304
34	Lyu Jy	2023	CC	Eng.	32	13	43.2	54	13	41	0	0	61	CR vs. CR + PRP	24
35	Wang R	2022	RCT	Chi.	157	99	45.2	256	0	256	0	0	6	CR vs. CR + CM	22
36	Lu Y	2023	CS	Eng.	152	62	43.0	246	0	157	89	0	0	CD + CR	/
**Tot.**					**1301**	**664**		**2222**	**247**	**1696**	**277**	**2**			**4651**
**Avg.**							**40.4**						**20.44**		**133**

***CD*** Core decompression; ***CR*** Ceramic-rod ***ESC*** enriched bone marrow stem cells ***ABG*** bone autografting ***AB*** autologous bone; ***PRP*** Platelet-Rich Plasma ***TCM*** Traditional Chinese Medicine

### Surgical data

Surgery duration averaged 54.52 ± 17.92 min (25–120 min), incision length 2.8 ± 1.0 cm (1–5 cm). Blood loss was 64.42 ± 22.77 ml (10–200 ml), hospitalization lasted 8.80 ± 3.16 days (4–14 days). Post-surgery, crutches were used for 2.95 ± 1.03 months (double support) and 7.07 ± 3.08 months (single support). BR utilization resulted in minimally invasive treatment with a short surgery, small incision, minimal blood loss, and a brief hospital stay.

### Follow-up results

Overall follow-up rate was 98.54%±4.57%, averaging 20.44 ± 10.41 months (3–73 months), stratified as follows: 34.63% for ≤ 1 year, 40.91% for ≤ 2 years, 11.34% for ≤ 3 years, 10.84% for ≤ 4 years, and 2.28% for ≤ 5 years.

**Fig. 5:**
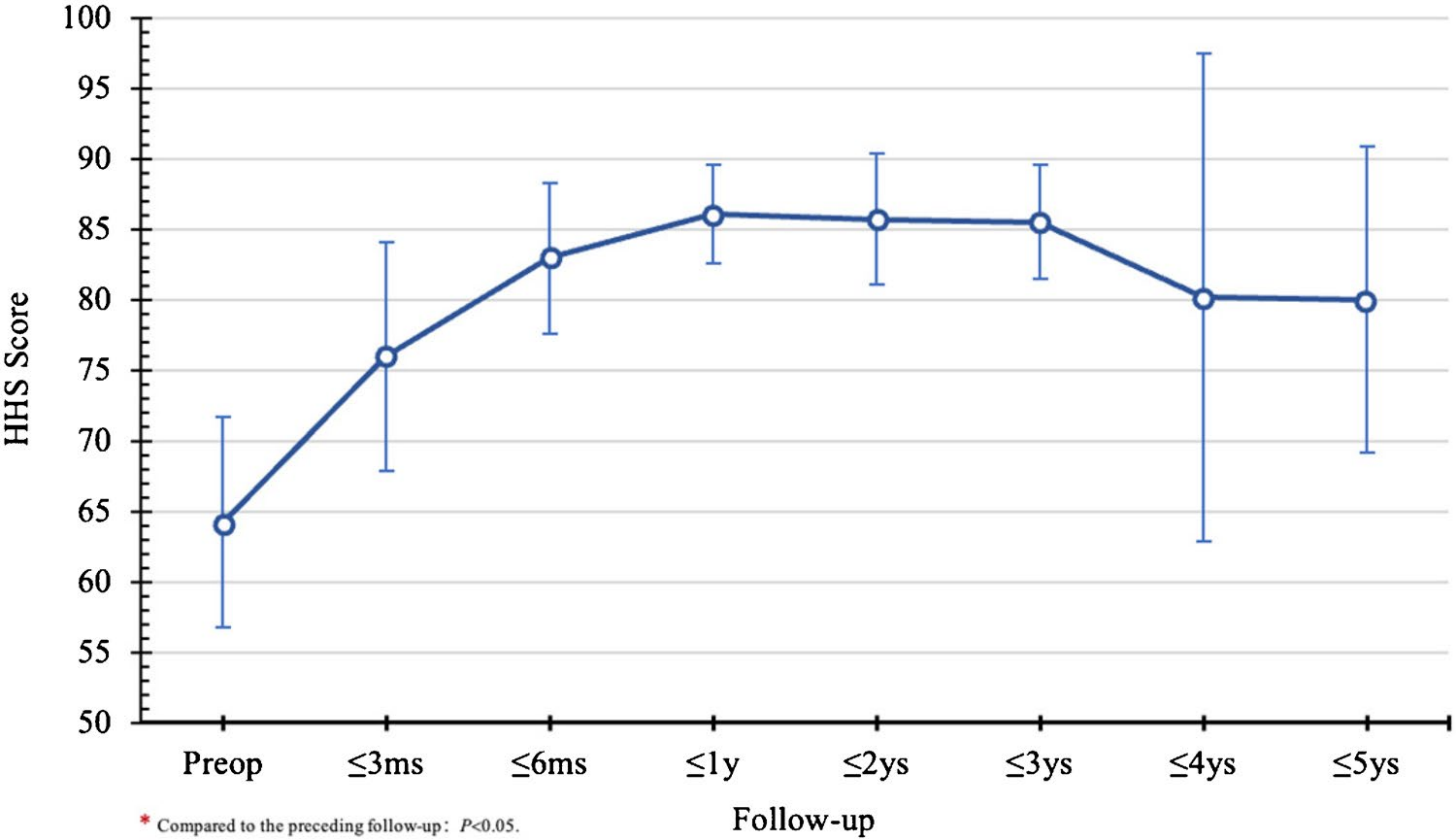
Trend of changes in HHS before and after surgery

**Fig. 6:**
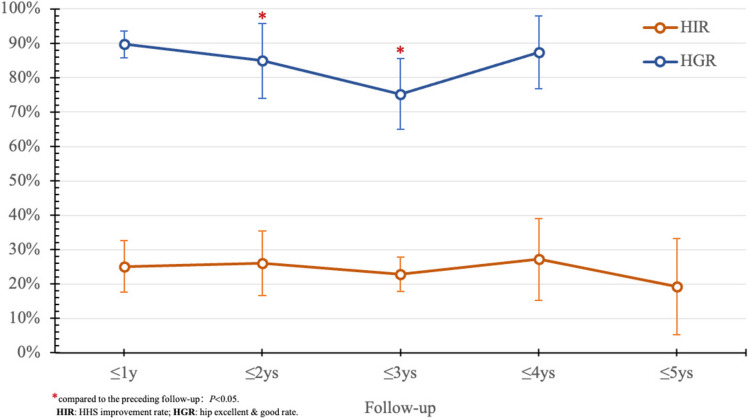
Trend of changes in HIR and HGR postoperative

Initial postoperative year showed rapid HHS increase, peaking at 82.40 ± 7.69 significant differences were noted at ≤ 3 months and ≤ 6 months compared to the preceding follow-up (*P* < 0.05). Subsequently, there was a gradual decline in HHS (Fig. 5), with no significant differences in the overall HIR (33.93%) observed across the follow-up periods. The HGR peaked in the first year and gradually decreased (Fig. 6), reaching an overall rate of 83.70% (Tables 2 and 3). BRs positively impacted symptom relief and functional recovery, stabilizing after one year.

**Table 2:** Statistical analysis of the extracted data

Follow-up	HHS	HIR	HGR	HSR	FSR	HRR
Preop.	64.23±7.46	—	—	—	—	—
≤ 3ms	76.01±8.12^*^	—	—	—	—	—
≤ 6ms	82.99±5.37^*^	—	—	—	—	—
≤ 1y	86.16±3.51	25.14%±7.51%	89.68%±3.97%	88.31%±5.31%	88.85%	30.43%±1.65%
≤ 2ys	85.77±4.64	26.02%±9.34%	84.88%±10.80%^*^	90.92%±6.62%	85.73%^*^	65.22%±3.54%
≤ 3ys	85.55±4.04	22.92%±5.02%	75.23%±10.31%^*^	85.09%±14.22%	77.04%^*^	4.35%±0.24%
≤ 4ys	80.26±17.31	27.20%±11.90%	87.30%±10.57%	82.50%±4.34%	75.20%	0
≤ 5ys	80.05±10.85	19.24%±13.90	—	80.19%±7.06%	85.19%	0
**Overall**	**82.40**	**24.11%**	**84.27%**	**85.35%**	**84.42%**	**5.42%**

***HIR*** HHS improvement rate; ***HGR*** Hip excellent & good rate ***HSR*** Femoral head stability rate ***FSR*** Femoral head stability rate ***HRR*** Hip replacement rate

*Compared to the preceding follow-up: *P*<0.05

**Table 3:** Meta-analysis of the extracted data ( Effect Size + 95%-CI)

Follow-up	HIR	HGR	HSR	HRR
≤ 1y	31.78%	86.92%	88.42%	7.58%
	(26.68–37.36%)	(77.91–92.6%)	(85.52–90.8%)	(4.55–12.37%)
≤ 2ys	35.42%	86.22%	91.03%	7.31%
	(25.2–47.2%)	(79.75–90.86%)	(87.22–93.78%)	(5.72–9.30%)
≤ 3ys	30.77%	73.36%	87.33%	5.33%
	(25.4-36.73%)	(67.11–79.26%)	(70.01–95.32%)	(2.02–13.36%)
≤ 5ys	30.77%	—	77.0%	0
	(21.52–46.77%)		(71.9-81.41%)	
≤ 6ys	—	—	88.42%	0
			(85.52–90.8%)	
**Overall**	**33.93%**	**83.70%**	**88.91%**	**7.97%**
	**(28.63–39.66%)**	**(78.9-87.6%)**	**(85.19–91.42%)**	**(6.06–10.42%)**

***HIR*** HHS improvement rate; ***HGR*** Hip excellent & good rate ***HSR*** Femoral head stability rate ***FSR*** Femoral head stability rate ***HRR*** Hip replacement rate

**Fig. 7:**
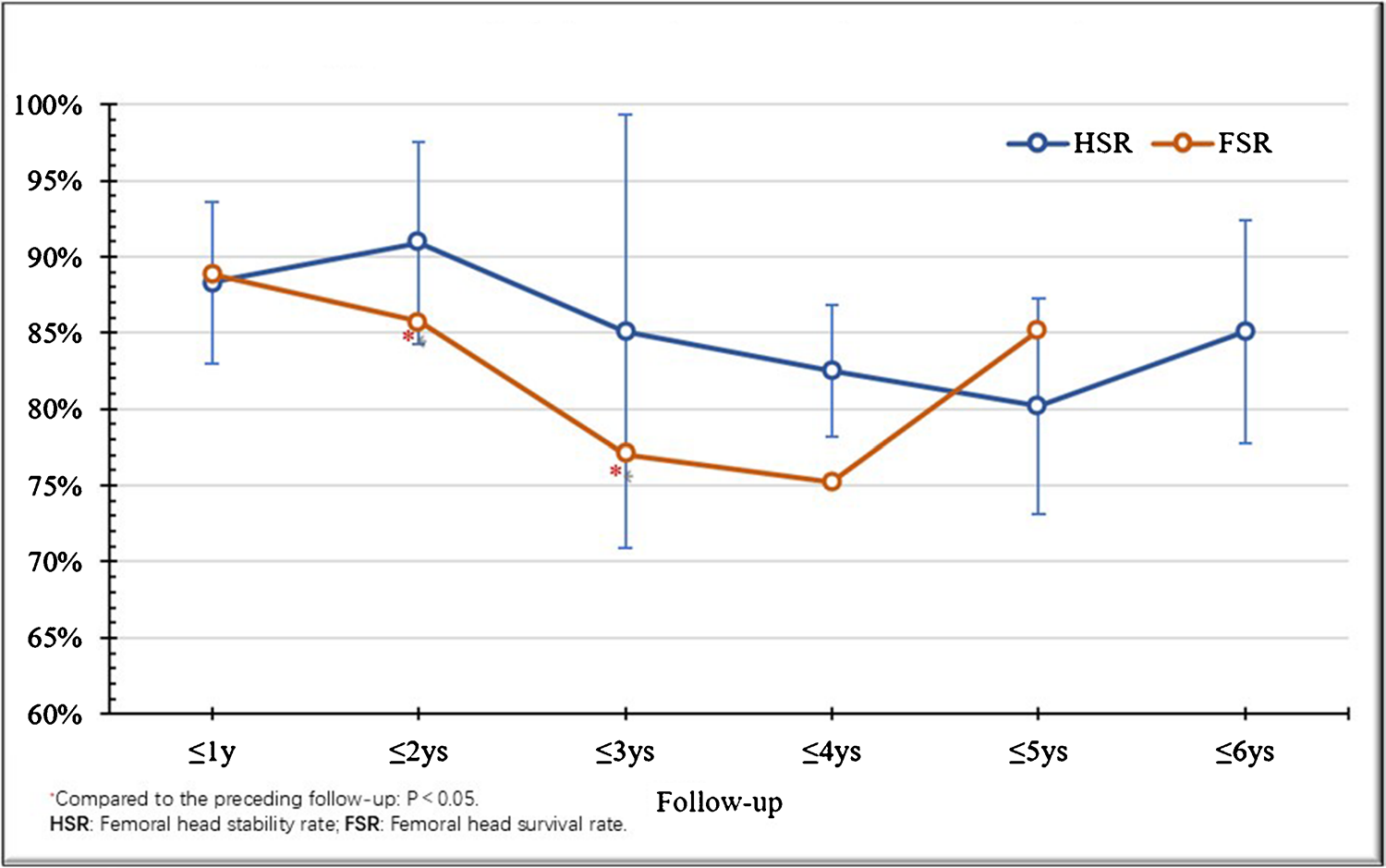
Trend of changes in HSR ang FSR postoperative

Radiological stability reached its maximum after 2 years, yielding an overall HSR of 88.91%. FSR declined in the initial four years, culminating in an 84.42% overall rate, with significant differences within the first three years (*P* < 0.05) (Fig. 7). The overall HRR was 7.97%, with 30.43% in the first year, 65.22% in the second year, and 4.35% in the third year; no replacements occurred thereafter. The efficacy of BRs was evident, achieving hip-preserving outcomes as early as 3 years postoperative.

**Fig. 8:**
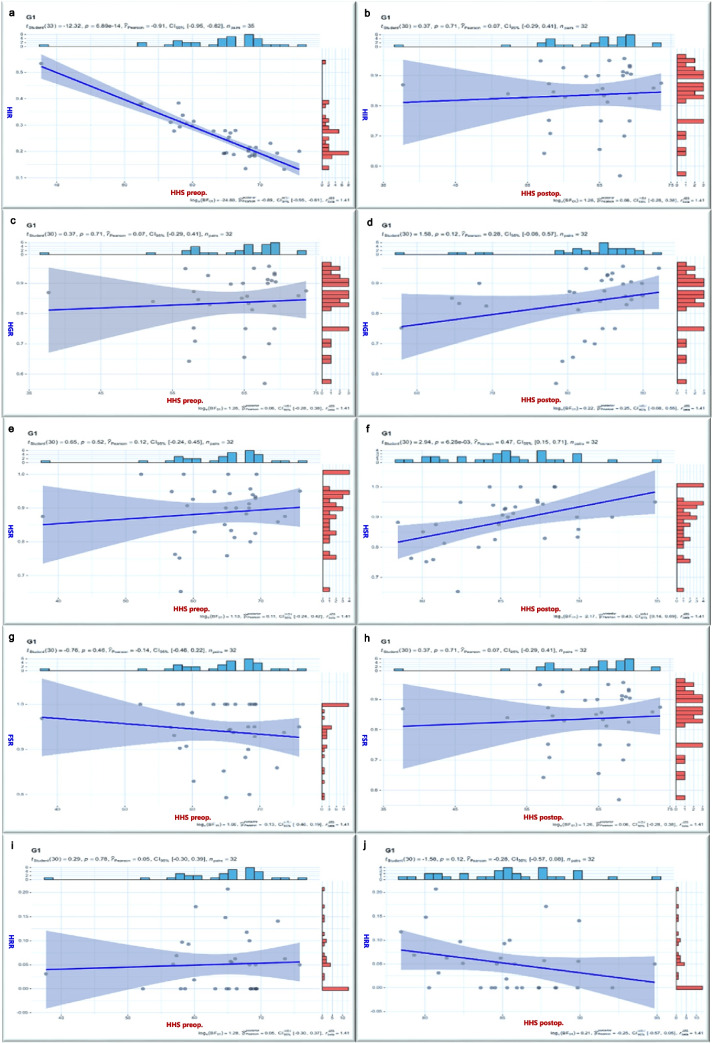
Correlation results between patient symptoms and efficacy indicators. ***HIR*** HHS improvement rate, ***HGR*** Hip excellent & good rate, ***HSR*** Femoral head stability rate, ***FSR*** Femoral head stability rate, ***HRR*** Hip replacement rate

**Fig. 9:**
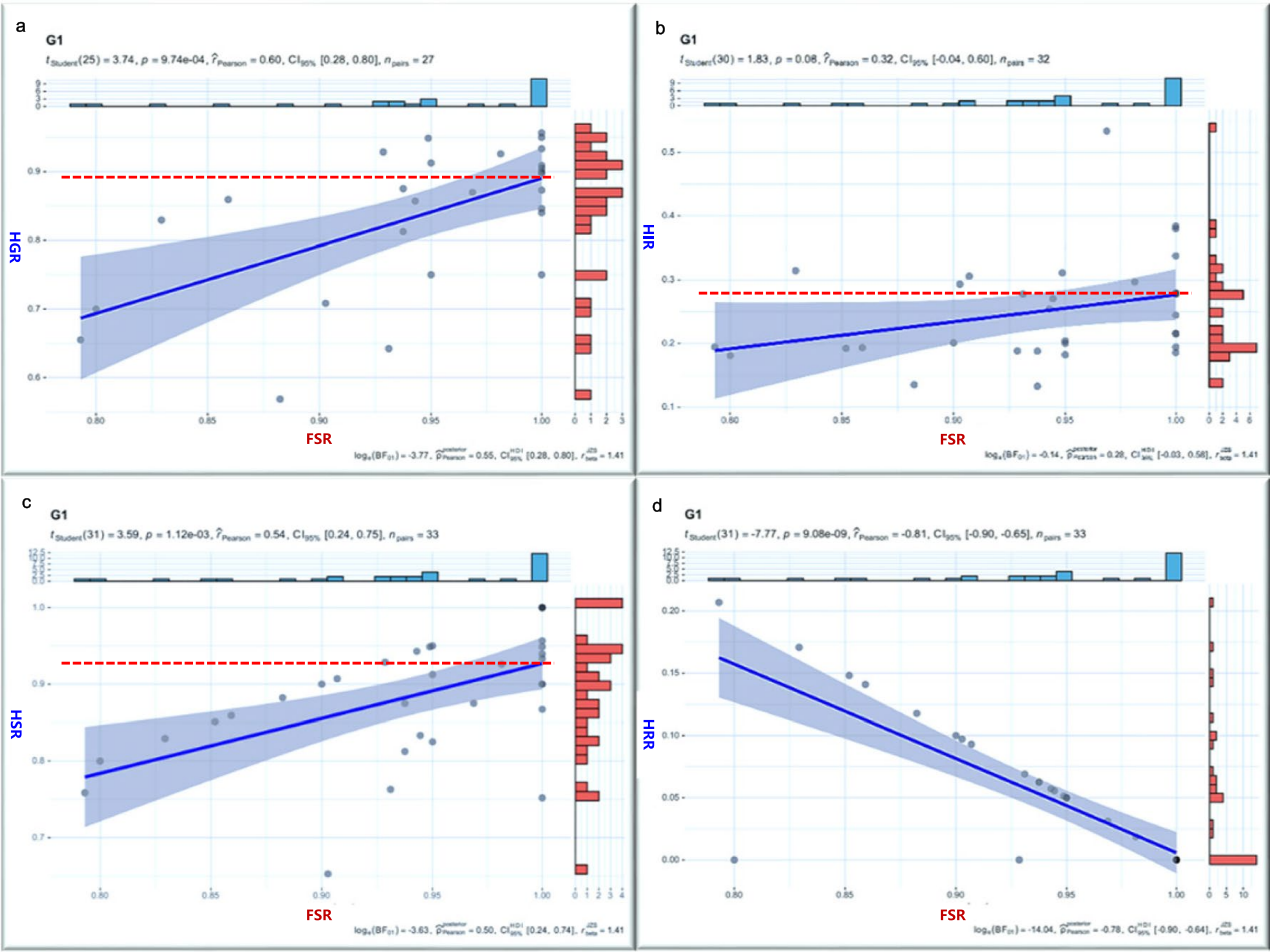
Correlation results between FSR and other therapeutic indicators. ***HIR ***HHS improvement rate,***HGR*** Hip excellent & good rate, ***HSR*** Femoral head stability rate, ***FSR*** Femoral head stability rate, ***HRR*** Hip replacement rate

In the correlation study, lower preoperative HHS significantly correlated negatively with the HIR (*r* = −0.912), unrelated to postoperative HHS. Postoperative HHS improvement correlated positively with a higher HGR (*r* = 0.600) and HSR (*r* = 0.473), unrelated to preoperative HHS. As postoperative HHS increased, FSR rose, and HRR decreased, irrespective of preoperative HHS (Fig. 8). FSR correlated positively with HGR, HSR (*r* = 0.599, *r* = 0.542, respectively), and negatively with HRR (*r* = −0.786,). Hip preservation success increased with overall HIR exceeding 27%, HGR approaching 90%, or HSR surpassing 90% (Fig. 9). Improvement in postoperative symptoms directly influenced clinical outcomes, emphasizing the critical importance of efficacy indicators in assessing hip-preserving outcomes.

### Comparative studies

#### BR for Stage II vs. Stage III of ONFH

In treating 150 hips in Stage II and 82 in Stage III with BRs (average age 42, mean follow-up 22.7 months), both groups showed over 28% increase in final HHS (*P* < 0.001). Stage II had significantly higher HGR (84.6%) and HSR (90.67%) compared to Stage III (69.7% and 58.54%, respectively) (*P* = 0.001). Disease progression in Stage II (9.33%) was significantly lower than in Stage III (41.46%) (*P* = 0.0001). The overall FSR in Stage II (96.0%) was higher than in Stage III (87.8%) (*P* = 0.019) [17]. The BRs technique exhibits evident efficacy in hip-preservation, with outcomes showing a positive correlation to the stage of the disease.

#### BR vs. Core Decompression (CD)

In a comparative study of Stage I-II patients, both CD and BR treatments were assessed, with 20 hips in each group (average age 35.4 years, mean follow-up 27.5 months). Significant improvements in HHS were observed for both groups, with the BR group showing a higher HIR (25.7% vs. 19.8%, *P* = 0.038), a greater HGR (95.0% vs. 70%, *P* = 0.046), and increased HSR (90% vs. 60%, *P* = 0.032) compared to the CD group [21]. These findings highlight the observed superiority of BRs over CD in terms of symptom improvement and successful hip preservation.

#### BR vs. Autologous Bone Grafting (ABG)

In two studies comparing BR treatment (55 hips) to ABG (51 hips) for stage I to III patients, with follow-up durations of 12 and 29.27 months, both groups showed a significant improvement in the final HHS. One study indicated that the BR group had higher final HHS and HIR than the ABG group (*P* < 0.05) and a hig

her HGR (*P* < 0.05) [26]. While the second study reported no significant differences [33]. Although not statistically significant, the BR group showed higher HSR and FSR. Moreover, the FSR for stage II patients was higher than for stage III patients (*P* < 0.05) (Table 4). These findings confirm that the BR technique significantly outperforms simple ABG in terms of symptoms improvement and hip preservation.

**Table 4:** Comparison of the therapeutic effects of ceramic rods and bone autografting

Treatment method		Literature 26		Literature 33	
		Group CR	Group ABG	Group CR	Group ABG
Case Number		30 Cases	30 Cases	20 Cases	16 Cases
		(33 Hips)	(34 Hips)	(22 Hips)	(17 Hips)
Patient ages		41.9 ± 9.8ys	43.3 ± 9.4ys	32.31 ± 6.52ys	31.54 ± 5.86ys
Follow-up		12 months		29.27 ± 3.56 months	
ARCO	I stage	14 Hips	17 Hips	—	—
	II stage	18 Hips	17 Hips	17 Hips	14 Hips
	III stage	—	—	5 Hips	3 Hips
HHS	Preop.	68.82 ± 6.65	67.06 ± 6.44	68.45 ± 3.93	67.81 ± 4.47
	Final	84.52 ± 5.41^*^	79.68 ± 6.41	83.59 ± 4.97	82.31 ± 5.38
HIR		18.57%^*^	15.84%	18.11%	17.61%
HGR		90.90%	70.60%	70.00%	68.75%
HSR		93.94%	90.91%	81.82%	76.47%
FSR		97.00%	91.20%	II:88.25%^*^	II:76.92%
				III:50.00%^*^	III:66.67%

***HIR*** HHS improvement rate; ***HGR*** Hip excellent & good rate ***HSR*** Femoral head stability rate ***FSR ***Femoral head stability rate ***HRR*** Hip replacement rate* CR group vs. ABG Group: *P*<0.05

#### BR in combination with Autologous Bone (AB)

In a study treating 40 stages II to III ONFH hips, either with BR alone (BR group) or in combination with AB (AB group), with 20 hips in each group, and an average age of 35 years, the mean follow-up was 27.34 months. Both groups demonstrated significant postoperative improvements in HHS compared to preoperative scores (*P* < 0.05). The BR group exhibited higher HHS at 12 months and final follow-up than the AB group (*P* < 0.05). However, the AB group had a higher HGR, and HSR (78% and 85%, respectively) than the BR group (70% and 80%, respectively) (*P* < 0.05). During follow-up, three cases in the BR group and two cases in the AB group underwent total hip arthroplasty. The FSR for stages II and III in the BR group (88% and 67%, respectively) were significantly lower than the AB group (94% and 75%, respectively) (*P* < 0.05) [35]. This suggests that BR in combination with AB may improve hip-preserving outcomes but with potential risks of worsening symptoms.

#### BR in combination with Enriched bone marrow Stem Cells (ESC)

In a study treating 29 hips with BR alone and 22 hips with BR in combination with ESC (average age 37.70 years, mean follow-up 24 months), the ESC group’s final HHS was higher than the BR group (*P* = 0.041). The respective success rates were 72.73% and 44.83% (*P* = 0.052), whereas failure rates were 4.5% and 17.2% (*P* = 0.340). Kaplan-Meier analysis did not show a significant difference in FSR (*P* = 0.203), but a trend indicated higher rates in the ESC group. Spearman rank correlation linked postoperative HHS and HRR [34]. This suggests that BRs in combination with ESC may contribute to symptom improvement, although there is insufficient evidence to support an enhancement of overall efficacy.

#### BR in combination with Platelet-Rich Plasma (PRP)

**Fig. 10:**
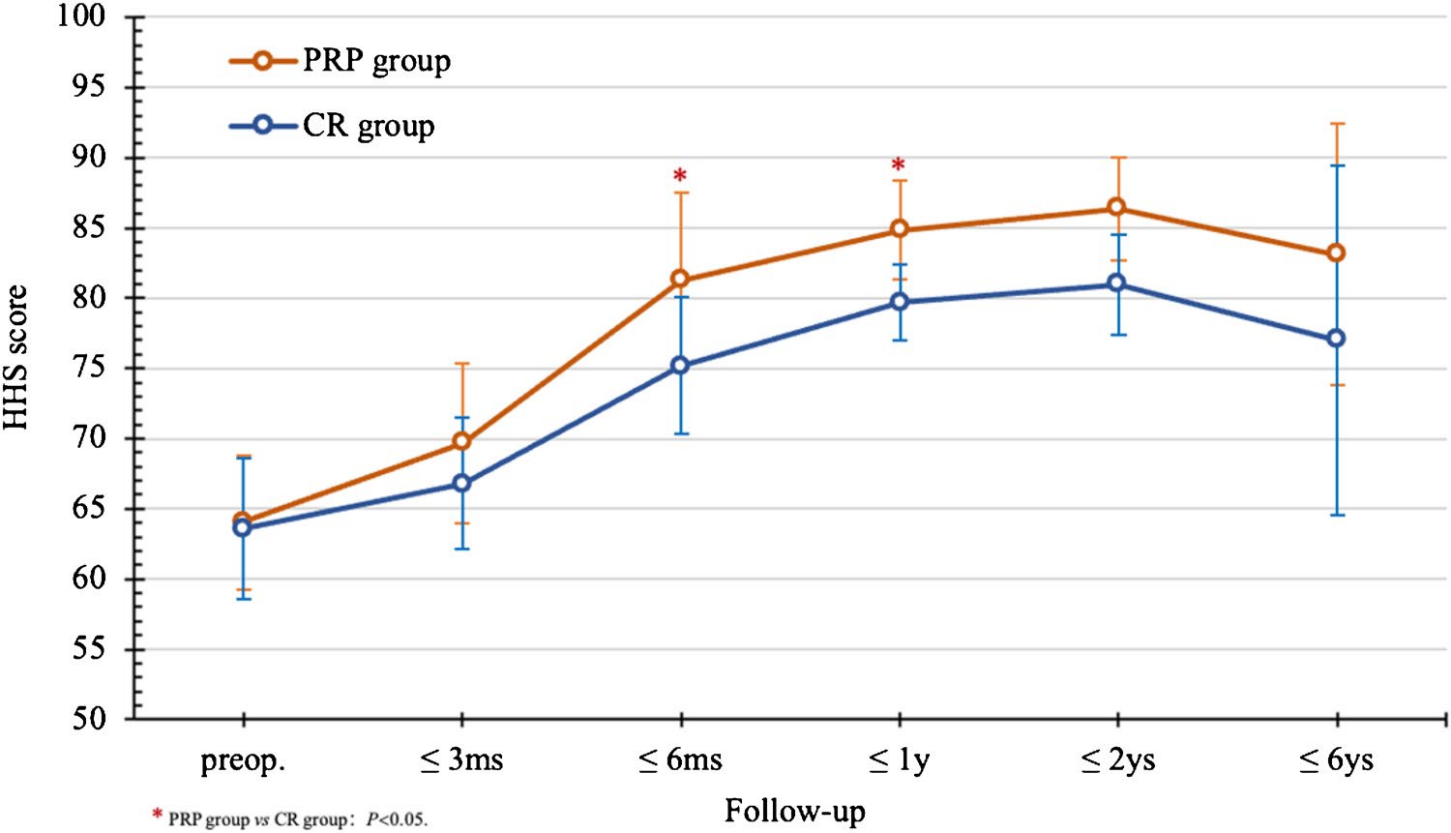
Comparison of HHS results between CR and PRP

**Fig. 11:**
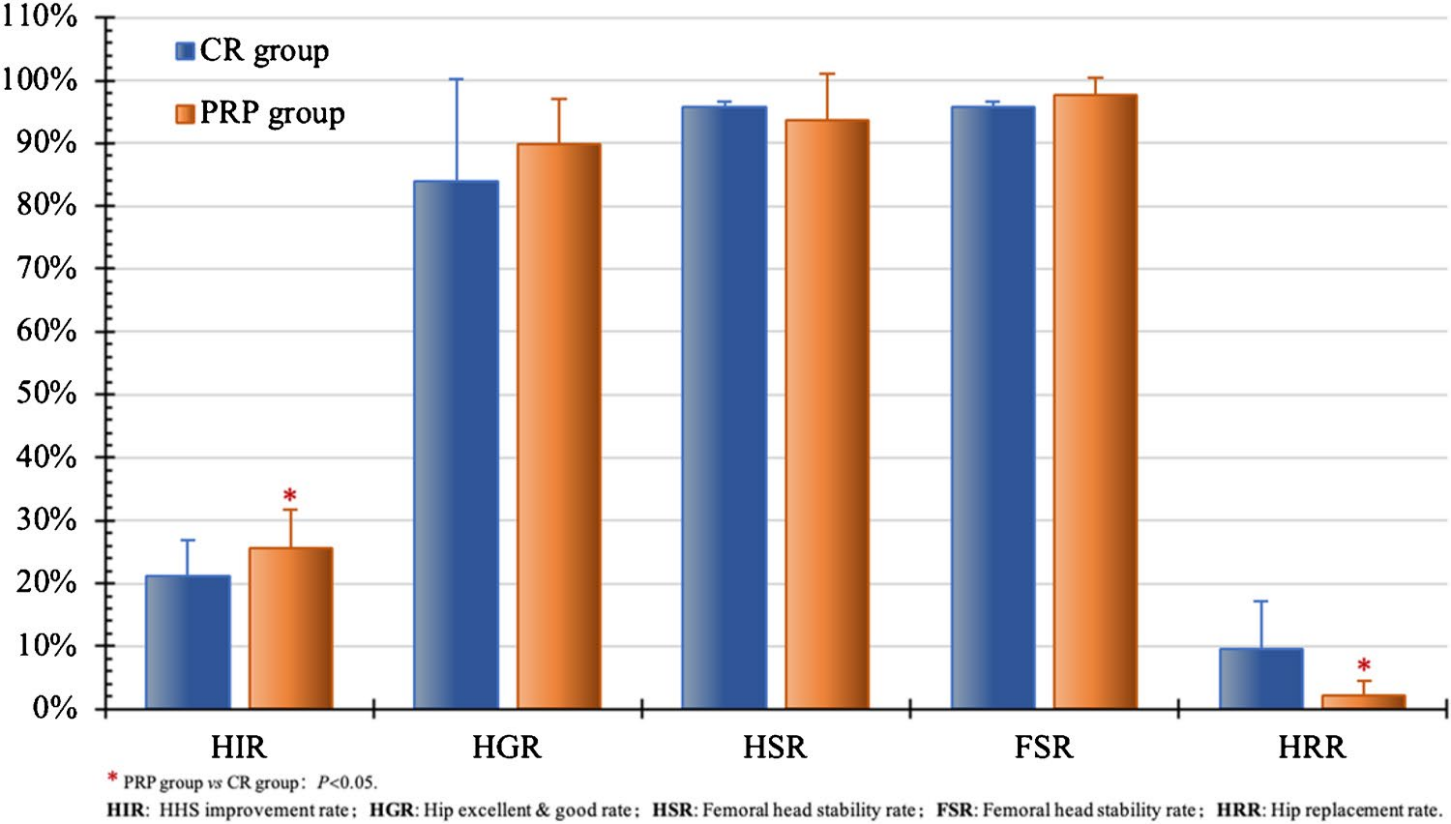
Comparison of efficacy between CR and PRP postop.(2 years)

In six studies [18, 20, 30, 32, 37, 38], 210 hips received BR treatment, and 221 hips received BR combined with PRP in stage I to II ONFH patients, averaging 42.67 years old with a mean follow-up of 25.22 months. In both groups, HHS peaked within two years, significantly improving at six months and one year compared to preoperative scores (*P* = 0.0012 and *P* = 0.0003, respectively), and the differences gradually diminished thereafter (Fig. 10). At three to 24 months, the PRP group showed higher HHS and HIR than the BR group (*P* < 0.05). The HGR, HSR, and FSR were comparable between groups, with a slightly higher HRR in the BR group (*P* = 0.0333) (Fig. 11). These findings suggest that the combination of BR with PRP contributes to the improvement of symptoms and diminishes the need for joint replacement.

#### BR in combination with Traditional Chinese Medicine (TCM)

**Fig. 12:**
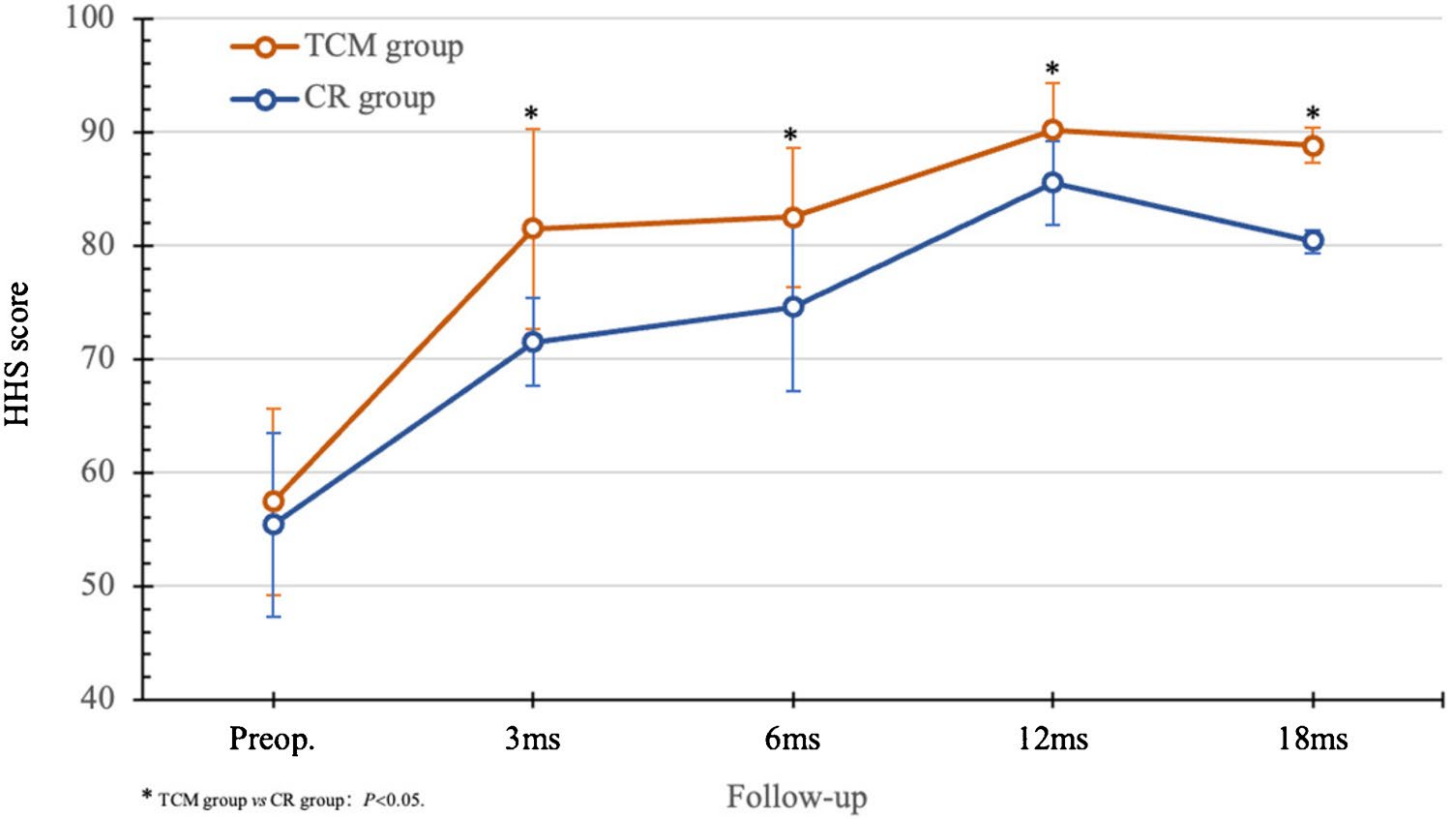
Comparison of HHS results between CR and TCM

**Fig. 13:**
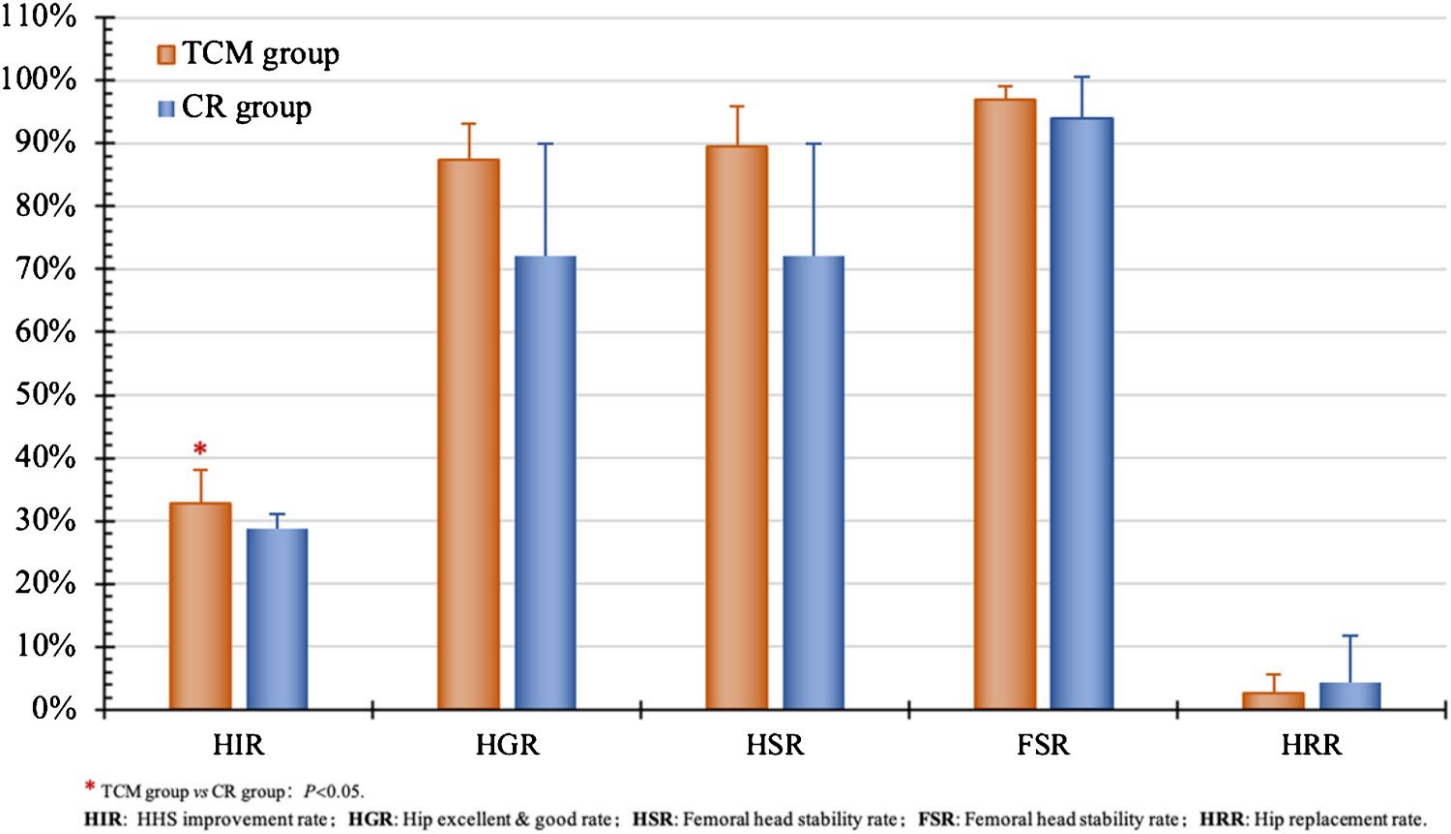
Comparison of efficacy between CR and TCM postop. (18 months)

In four studies [14, 19, 25, 39], BRs were investigated for treating 170 hips, and a combination of BRs and TCM was used for 207 hips in ONFH management. Average age was 38.15 years, with a mean 12.69-month follow-up. HHS evaluation showed both groups peaked within the first year, maintaining stability thereafter. Over the three to 18 months follow-up period, the TCM group demonstrated higher HHS and a superior final HIR compared to the BR group (*P* < 0.05) (Fig. 12). However, no significant distinctions were observed in the final HGR, HSR, HRR and FSR (Fig. 13). These findings suggest that the incorporation of TCM alongside BRs may offer benefits in terms of symptom improvement, yet no conclusive evidence supports an enhancement in overall efficacy.

## Discussion

The BR technique introduces an innovative approach utilizing porous structure-mediated revascularization for ONFH treatment, effectively connecting the blood supply between the greater trochanter and femoral head. This connection bridges two previously independent vascular systems, fostering the reconstruction of blood circulation within the necrotic area. During the human developmental process, the formation of the epiphysis and blood supply in the femoral head occurs around four months after birth, while the greater trochanter undergoes development between four to six years old. This significant developmental time gap results in non-communicating structures. The channel established during the BR procedure relieve intraosseous high pressure, providing relief from symptoms. Simultaneously, the expandable reamer [40] allows precise removal of necrotic bone, breaking down the sclerotic rim, promoting vascular ingrowth and providing a three-dimensional repair space. The implanted bioceramic material induces tissue regeneration, contributing to biomechanical reconstruction and reviving the necrotic femoral head [2].

This study not only utilizes meta-analysis to assess literature bias and sensitivity but also evaluates academic and practical value as well as field attention through literature impact factors, citation rates, and number of view, ensuring the accuracy and effectiveness of information and data. Comprehensive results demonstrate that BRs play a positive role in symptom relief and functional recovery, closely associated with effective intraosseous decompression, reduction of the lesion, and inflammation reduction. Evidence derived from EBM substantiates the safety and significant clinical effectiveness of the BR technique. In the initial postoperative year, there was a peak in symptom improvement and the excellent and good rate. The stability rate remained constant in the second year, with no joint replacements observed after three years. By the fourth year, the survival rate experienced a decline to its lowest point, but the overall survival rate surpassed 84%. The postoperative improvement of symptoms is crucial to assess efficacy, and the indicators play a guiding role in evaluating the hip-preservation outcome. The success rate of hip preservation is significantly elevated when achieving an overall HHS improvement exceeding 25%, an excellent and good rate approaching 90%, or a stability rate surpassing 90%. The author’s experience suggests that severe preoperative joint cartilage damage, extensive osteochondral separation, and premature postoperative weight-bearing are the main reasons for treatment failure, underscoring the importance of considering these factors when deciding whether to proceed with the surgery.

Various treatments exist ONFH, and opinions on their efficacy are diverse. An analysis of 32 studies on CD highlights the significance of preoperative staging as a pivotal determinant of treatment outcomes. Specifically, the femoral head survival rate in Stage III cases is reported to be merely 27.44% [41]. With an overall five-year follow-up rate of 77%, our study demonstrated the significant therapeutic role of BRs. A review of ten studies comparing different bone grafting treatments revealed that bone graft combined with bone marrow stem cells, and biomaterials graft emerged as effective therapeutic methods for ONFH, especially in improving symptoms, halting disease progression, and preventing joint replacement. Conversely, core decompression exhibited the least favorable outcomes [42]. Bioactive substances are preferred for ONFH treatment due to the dual benefits of relieving symptoms, halting disease progression, thus reducing the likelihood of early joint replacement [42]. An analysis of 17 randomized controlled studies found that stem cell therapy is superior to CD, ABG, and non-surgical treatment in preventing disease progression [43]. Additionally, a 30-year follow-up of bilateral steroid-induced early-stage cases demonstrated that joint replacement rates were three times lower with stem cell therapy compared to CD [44]. In a cohort of 35 non-collapse cases, CD, ESC, and PRP combined treatment resulted in a 93% non-collapse rate during a three-year follow-up [45]. A comprehensive analysis of 14 randomized controlled trials revealed that, 24 months after surgery, stem cell treatment exhibited notable improvements in symptoms and replacement rates compared to CD. However, it did not prove effective in preventing the progression of necrosis in advanced cases. However, it did not prove effective in preventing the progression of necrosis in advanced cases [46]. A systematic review of 15 studies suggested that vascularized fibular grafting demonstrates superior efficacy compared to CD and non-vascularized fibular grafting, particularly in the early stages of the disease among young patients. Nevertheless, additional research is warranted to establish a consensus regarding its effectiveness [47].

The BR-mediated technique not only provides an effective blood supply and a three-dimensional repair space but, more importantly, guides vascular regeneration and repairs necrotic areas through a controllable porous micro-structured material. The combination of BRs with AB can enhance overall efficacy; however, it comes with the risk of symptoms worsening. Conversely, the efficacy of ABG alone is comparatively lower than that of BRs, as ABGs undergo necrosis in ischemic conditions, consequently exacerbating the symptoms. In contrast, the degradation of inorganic bioceramics primarily occurs through liquid dissolution. Consequently, when subjected to ischaemic conditions, BR experiences a reduced degradation rate, thereby maintaining its structural integrity and minimizing the risk of collapse. Furthermore, in certain cases, postoperative imaging around the two-month mark depicts a low-density band at the bioceramic-host bone interface. This is frequently misinterpreted as bone absorption or progression of necrosis, while in reality, it represents an overlay of incompletely mineralized new bone and partially degraded interface material, gradually disappearing over time. Combining ESC, PRP, or TCM with BR is beneficial for symptom relief, but evidence is insufficient to prove their role in hip preservation, indirectly demonstrating the dominant role of BR in the treatment of ONFH. In contrast to existing treatments, BR presents several advantages, including reduced operation time, minimized bleeding, smaller incisions, procedural simplicity, and greater feasibility for widespread adoption. The surgical procedure preserves the overall anatomical form and structure, while BR undergoes complete degradation and absorption, mitigating the challenges associated with joint replacement in cases of treatment failure.

The treatment of femoral head necrosis with BR mediated revascularization technique involves directing normal blood flow into the necrotic area. This advances traditional hip-preserving treatment to a more proactive “head-saving” approach. Evidence-based medicine confirms the high safety, reliable efficacy, minimal trauma, and simplicity of the technique, indicating a broad spectrum of potential applications.

## Data Availability

Data is provided within the manuscript.
